# Correlation between Corneal Endothelial Cell Loss and Location of Phacoemulsification Incision

**Published:** 2011-01

**Authors:** Hamid Gharaee, Abbas Kargozar, Ramin Daneshvar-Kakhki, Maria Sharepour, Samira Hassanzadeh

**Affiliations:** 1 Department of Ophthalmology, Mashhad University of Medical Sciences, Mashhad, Iran; 2 Paramedical College, Mashhad University of Medical Sciences, Mashhad, Iran

**Keywords:** Endothelial Cell Loss, Incision, Phacoemulsification

## Abstract

**Purpose:**

To assess the relationship between corneal endothelial cell loss after phacoemulsification and the location of the clear corneal incision.

**Methods:**

A total of 92 patients (92 eyes) with senile cataracts who met the study criteria were included in this cross sectional study and underwent phacoemulsification. The incision site was determined based on the steep corneal meridian according to preoperative keratometry. Endothelial cell density was measured using specular microscopy in the center and 3 mm from the center of the cornea in the meridian of the incisions (temporal, superior, and superotemporal). Phacoemulsification was performed by a single surgeon using the phaco chop technique through a 3.2 mm clear cornea incision. Endothelial cell loss (ECL) was evaluated 1 week, and 1 and 3 months postoperatively.

**Results:**

At all time points during follow-up, ECL was comparable among the 3 incision sites, both in the central cornea and in the meridian of the incision (P > 0.05 for all comparisons). However, 3 months postoperatively, mean central ECL with superior incisions and mean sectoral ECL with temporal incisions were slightly higher. Superotemporal incisions entailed slightly less ECL than the other 2 groups. Overall, one month after surgery, mean central ECL was 10.8% and mean ECL in the sector of the incisions was 14.0%. Axial length and effective phaco time (EFT) were independent predictors of postoperative central ECL (P values 0.005 and < 0.0001, respectively).

**Conclusion:**

A superotemporal phacoemulsification incision may entail less ECL as compared to other incisions (although not significantly different). The amount of central ECL may be less marked in patients with longer axial lengths and with procedures utilizing less EFT.

## INTRODUCTION

Since the first phacoemulsification procedure performed by Kelman^1^ in 1967, corneal endothelial cell loss (ECL) remains a serious concern in cataract surgery.^2^ Damage to the corneal endothelium is influenced by various pre- and intraoperative factors.^2^ In phacoemulsification, the amount of ECL determines final postoperative corneal transparency and to some extent visual acuity. Thus, attempts to protect the corneal endothelium and to minimize their damage can play an important role in improving quality of life in patients with cataracts.^3^,^4^

The location of corneal access (the incision site) can influence ECL.^5^ According to previous studies, using a scleral tunnel incision, there is no significant difference between superior and temporal incisions selected based on preoperative keratometry.^2^ However, in order to minimize postoperative astigmatism, the location of corneoscleral access may vary;^2^ in some cases, the surgeon must perform superotemporal incisions. In the current study we compared the amount of ECL after clear cornea phacoemulsification using superior, temporal, or superotemporal incisions. In addition, we evaluated factors which may influence the amount of postoperative ECL in the central cornea and in the meridian of the incisions.

## METHODS

A total of 92 patients (92 eyes) were included in this cross-sectional study according to the criteria presented in table 1.

Preoperatively, all patients underwent a complete ophthalmologic examination. Axial length was measured by A-scan ultrasonography (Optikon 2000 SpA, Optikon, Rome, Italy). Before surgery, the site of the clear cornea incision was selected on the steep meridian of the cornea based on keratometry, which was assessed by an autorefracto-keratometer (KR 8800, Topcon, Tokyo, Japan) considering the patient’s refraction. Only eyes in which the steepest meridian of the cornea was within 20° of the 0°, 180°, or 90° axes or within 10° of the 45° (for the left eye) or 135° (for the right eye) axes were selected for the purpose of the study.

Preoperatively, endothelial cell density (ECD) was measured using a specular microscope (Tomey EM3000, Tomey Corporation, Miami, FL, USA) in the center of the cornea and 3 mm from the center in the meridian of the incision. For each eye, three measurements were performed and the average value was calculated; at least 100 cells were evaluated in each measurement. ECD measurement was repeated 1 week, 1 month and 3 months postoperatively. In order to measure ECL we used the following formula:

$$\text{ECL}\%=\frac{\text{preoperative ECD}-\text{postoperative ECD}}{\text{preoperative ECD}}\times 100$$

Phacoemulsification was performed using the Fritz Ruck Pentasys 2 (Ophthalmologische Systeme GmbH, Eschweiler, Germany) by a single expert surgeon, utilizing a bimanual phaco chop technique through a 3.2 mm clear cornea incision. For all patients, a hydrophilic acrylic posterior chamber intraocular lens (Kontur AB, Medicontur Ltd, Zsámbék, Hungary), was implanted. No sutures were placed for any of the eyes.

For data analysis, SPSS statistical software version 13 was used. Since all parameters were normally distributed and the variances were homogenous, comparison among groups was performed using the ANOVA and comparison within groups was performed by paired t-tests. Correlations among parameters were evaluated utilizing Pearson tests. Ultimately, we used stepwise multiple regression to detect independent influential factors.

## RESULTS

Ninety-two patients (92 eyes) including 45 men (48.9%) and 47 women (51.1%) with mean age of 53.0±4.5 (range, 42 to 60) years were allocated to three groups according to the incision site: temporal (24 eyes, 26%), superior (34 eyes, 37%) and superotemporal (34 eyes, 37%). Baseline patient data are presented in table 2.

### ECL in the central cornea

**Figure 1:**
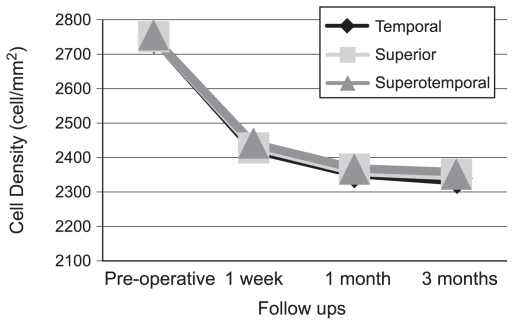
Changes in mean central endothelial cell density with time in each study group.

Overall, mean central ECD was 2542.9±194.0 (range, 2004 to 2897) cells/mm^2^ preoperatively. No significant difference was observed among the three study groups in terms of central ECD preoperatively or at any follow-up period (Table 3). Figure 1 depicts changes in mean central ECD with time in each incision group.

Overall, mean ECL in the center of the cornea was 10.8% one month postoperatively. The amount of ECL at different follow-up intervals is presented in table 4.

Although mean central ECL was not significantly different among the three groups (table 4), it was significantly reduced within each of them during follow-up (P<0.001 for all comparisons, paired sample t-test).

### ECL in the meridian of the incision

Mean ECD in the meridian of the incision was 2749.0±224.6 cell/mm^2^ preoperatively (range, 2145 to 3345 cell/mm^2^). No significant difference was detected among the study groups preoperatively or at any follow-up interval (table 3).

Overall, mean ECL in the meridian of the incision was 14.0% one month postoperatively. The amount of ECL at different follow-up intervals is presented in table 4. Mean ECL in the meridian of the incision was not significantly different among the three groups (table 4), but was significantly reduced within all study groups throughout the follow-up period (P<0.001 for all comparisons, paired sample t-test). Three months postoperatively, the amount of ECL in the temporal incision group was slightly larger than in the other incision groups (14.9%).

In all three groups, mean central ECL was statistically lower than mean ECL in the meridian of the incision at all follow-up intervals (P<0.001, single sample t-test).

### Factors Influencing ECL

Three months postoperatively, a significant correlation was observed between mean ECL in the center of the cornea and axial length, operative time, and effective phaco time (EFT). However, there was no correlation between any of the study parameters and ECL in the meridian of the incision (Table 5).

To determine independent factors related to central ECL, we entered the abovementioned parameters in a stepwise multiple regression model. The results showed that shorter axial length and higher EFT were independent factors influencing ECL in the central cornea (P=0.005 and P<0.001, respectively).

## DISCUSSION

In recent years, various techniques have been employed in cataract surgery. The trend in modern cataract surgery is to minimize side effects, such as surgical trauma, corneal burn and loss of endothelial cells, and to reduce the size of the access area to minimize postoperative astigmatism.^6^,^7^

Phacoemulsification is a safe and effective procedure and represents the gold standard for cataract surgery.^6^ Nevertheless it is still associated with trauma.^6^ One of the complications of trauma is reduction in corneal ECD. In this study, we assessed the effect of different incision sites on corneal ECL postoperatively, and compared ECL in various incision sectors and also the central cornea.

According to our results, mean central ECLs, 1 week, 1 month, and 3 months postoperatively were 8.12%, 10.85%, and 10.44% respectively, and was comparable with various incisions. Three months after surgery, mean sectoral ECL was comparable among the study groups at 14.9% for temporal incisions, 14.5% for superior incisions, and 14.4% for superotemporal incisions.

Previous studies have reported different amounts of ECL (4.3%,^8^ 6.2%,^9^ 11.8%,^3^ and 18.3%^10^), which may be due to various factors such as different surgical technique, incision type and location, and cataract density. The amount of ECL in our patients was higher than that of previous reports. The reason may be that all of our patients had nuclear sclerosis of 3+ to 4+ requiring greater EFT and hence larger ECL.^5^,^11^ We performed the operation through a 3.2 mm incision on the clear cornea which was expected to minimize ECL.^12^

Similar to the study by Walkow et al^2^, we selected the incision site according to preoperative keratometry. They used a superior scleral tunnel for cataract removal and their results showed ECL of 8.5% at 12 months postoperatively. This figure was 11.9% in the temporal quadrant and 11.4% in the superior quadrant. Other reports^13^ have shown that mean ECL for temporal clear cornea incisions is more than that of superior scleral tunnel incisions. In concordance with the results of Walkow et al, we found that mean ECL was not significantly different between superior and temporal incisions (both in the corneal center and in the meridian of the incision).

At 3 months, mean ECL in the central cornea for all incision types was slightly less than mean central ECL at 1 month. This finding may be due to migration of peripheral endothelial cells into the central cornea to compensate for the loss.^2^ We also found that for all incision sites, mean ECL in the sector of incision was significantly more than that of the central cornea, which may be due to proximity to the phaco handpiece.^14^ Our results showed that although mean ECL, both in the corneal center and incision sector, was not significantly different among the three incision groups, mean ECL was slightly less in the superotemporal group than in patients who underwent the operation through superior or temporal incisions. This was true for all postoperative follow ups. We did not find any reports that support these results, but it seems that superotemporal incisions may have a lower risk for surgical trauma and ECL in patients.

In agreement with previous reports^2^,^8^ we found that 3 months postoperatively, shorter axial length and higher effective phaco time (EFT) were independent predictors for ECL in the central cornea. Walkow et al^2^ reported that a greater distance between the phaco tip and the endothelium in longer eyes, or greater relative variations in axial length compared to anterior chamber depth can produce such findings. Other studies have reported other factors related to ECL, such as the amount of ultrasound energy and cataract density.^5^

In summary, ECL seems to be comparable after phacoemulsification using different incision sites and the choice of incision may be made based on preoperative corneal keratometry. Postoperative central ECL is associated with with shorter axial lengths and higher EFT.

## Figures and Tables

**Table 1:** Inclusion and exclusion criteria

Inclusion criteria	Exclusion criteria
Age range, 40 to 60 years	Diabetic patient
Steep corneal meridian at 0°, 180° or 90° ± 20°, and 45° or 135° ± 10°	KR>48 D or <40 D AL>28 mm or <23 mm ECD<1500 cell/mm^2^ preoperatively
NS 3+ to 4+	Complicated cataract surgery
Senile cataract	History of intraocular surgery or ocular trauma
Uncomplicated phacoemulsification	Pseudoexfoliation or corneal endothelial dystrophy

NS, nuclear sclerosis; KR, keratometry reading; AL, axial length; ECD, endothelial cell density

**Table 2:** Baseline patient data

Factor	mean± SD	Min	Max
Age (years)	53.04±4.53	42	60
Axial length (mm)	24.60±0.68	23.2	26.1
Operation time (min)	6.35±0.86	5	9
EFT (sec)	6.36±1.66	3	10
Central ECD (cells/mm^2^)	2542.9±194.03	2004	2897
ECD (temporal)	2547.7±248.03	2145	3238
ECD (superior)	2748.9±206.9	2245	3254
ECD (superotemporal)	2751.3±231.01	2321	3345

EFT, effective phaco time; ECD, endothelial cell density; SD, standard deviation; Min, minimum; Max, maximum

**Table 3:** Mean ECD (cells/mm^2^) in the central cornea and the meridian of the incision

Incision group	Central cornea				Meridian of the incision			
	
	Pre op	1 week later	1 month later	3 months later	Pre op	1 week later	1 month later	3 months later
Temporal	2574.4±222.9	2363.2±217.1	2297.1±207.9	2306.3±206.8	2745.5±248.0	2431.8±252.1	2358.9±242.3	2338.2±262.8

Superior	2536.8±181.3	2322.2±173.7	2257.3±182.2	2268.9±187.2	2751.4±231.1	2439.0±217.3	2370.6±221.7	2357.4±228.7

Supratemporal	2526.8±187.9	2332.4±187.7	2258.1±201.1	2267.5±200.0	2748.9±206.9	2429.0±297.5	2365.0±303.9	2352.4±303.6

P-value*	0.643	0.715	0.701	0.72	0.95	0.92	0.95	0.945

Pre op, preoperatively; SD, standard deviation; ECD, endothelial cell density

* ANOVA

**Table 4:** Mean ECL (%) in the central cornea and sector of incision in different incision groups

Incision group	Central cornea			Sector of incision		
	
	1 week later	1 month later	3 months later	1 week later	1 month later	3 months later
Temporal	8.22±2.23	10.78±2.34	10.40±2.59	11.50±2.61	14.10±3.13	14.90±4.46

Superior	8.45±2.30	11.04±2.49	10.60±2.94	11.80±5.93	14.10±6.36	14.50±6.46

Supratemporal	7.72±2.10	10.70±2.78	10.32±2.84	11.40±1.93	13.90±2.60	14.40±3.14

P-value	0.379	0.849	0.922	0.92	0.97	0.92

**Table 5:** Correlation between study parameters and mean ECL 3 months postoperatively

Parameter	Corneal center		Meridian of incision	
	
	Pearson co-eff	P-value	Pearson co-eff	P-value
Age	−0.080	0.448	0.017	0.871

Axial length	−0.416	<0.001	0.116	0.272

EFT	0.540	<0.001	−0.016	0.879

Operation Time	0.161	0.044	−0.141	0.180

ECL, endothelial cell loss; EFT, effective phaco time; Pearson co-eff, Pearson coefficient
